# Clinical efficacy and safety of perampanel monotherapy as primary anti‐seizure medication in the treatment of pediatric epilepsy: A single‐center, prospective, observational study

**DOI:** 10.1002/epi4.13043

**Published:** 2024-09-18

**Authors:** Yanxia Gu, Yue Li, Wei Li, Feng Chen, Chunfeng Wu, Jing Chen

**Affiliations:** ^1^ Department of Neurology Children's Hospital of Nanjing Medical University Nanjing China; ^2^ Department of Pharmacy, Pharmaceutical Sciences Research Center Children's Hospital of Nanjing Medical University Nanjing China; ^3^ Department of Clinical Research Center Children's Hospital of Nanjing Medical University Nanjing China

**Keywords:** efficacy, epilepsy, monotherapy, pediatric, perampanel (PER), safety

## Abstract

**Objective:**

To assess the efficacy and safety of perampanel (PER) as primary monotherapy in patients aged 4–18 years old with epilepsy.

**Methods:**

A single‐center, prospective, observational study was conducted from October 2021 to October 2023, to evaluate PER monotherapy's efficacy and safety as initial therapy for pediatric epilepsy. Changes in seizure frequency, safety, and retention rate were observed at 3, 6, 9, and 12 months after initiating PER primary monotherapy.

**Results:**

A total of 124 children aged 4–15 years (mean age = 8.25 ± 2.50 years) were included in the Analysis Sets. The retention rates at 3, 6, 9, and 12 months were 88.71% (110/124), 84.68% (105/124), 78.26% (90/115), and 71.58% (68/95), respectively. Seizure freedom rates at 3, 6, 9, and 12 months were 85.45%, 79.09%, 76.24%, and 75.31%, respectively. The responder rates (≥50% but <100%) at the same endpoints were 9.09%, 14.55%, 12.87%, and 7.41%, respectively. Seizure freedom rate of PER was independent of age at PER initiation, seizure onset age, gender, baseline frequency, seizure types, and family history of epilepsy (*p* > 0.05) but associated with duration of treatment (*p* = 0.001) and maintenance dose (*p* = 0.022). Additionally, 124 patients were included in the safety analysis set. The overall adverse event rate was 38.71% (48/124), with irritability (19 cases, 15.32%) and dizziness (18 cases, 14.52%) being the most common adverse effects. One patient discontinued PER monotherapy within 1 month due to unbearable itching of the skin.

**Significance:**

PER monotherapy as the primary anti‐seizure medication (ASM) for pediatric epilepsy demonstrates high efficacy and safety in real‐world clinical treatment. Patients who respond well to this drug and adhere to long‐term treatment can achieve favorable seizure control. Furthermore, patients achieving seizure freedom with a relatively lower dose can opt for the same dose as the maintenance dose.

**Plain Language Summary:**

This study provided the efficacy and safety of PER monotherapy as the primary ASM for Chinese pediatric epilepsy. In total, 124 patients took part. The seizure freedom rates were over 70% at different observation points (OPs), along with a retention rate of 71.58% at the 12‐month OP. Most of adverse effects observed were mild to moderate.

**Key Points**

- Seizure freedom rates were over 70% at different OPs, along with a retention rate of 71.58% at the 12‐month OP.
- The overall adverse events rate was 38.71%, with irritability (19 cases) and dizziness (18 cases) being the most common adverse effects.
- PER monotherapy as the primary ASM for pediatric epilepsy demonstrates high efficacy and safety.
- Patients who respond well to this drug and adhere to long‐term treatment can achieve favorable seizure control.
- Patients achieving seizure freedom with a relatively lower dose can opt for the same dose as the maintenance dose.

## 1 INTRODUCTION

In the realm of pediatric epilepsy, inadequate seizure control imposes significant life and psychological burden on patients and their families.^1^ According to the results of the 7th National Census, nearly 10 million people in China are afflicted with epilepsy, with approximately 400 000 new cases emerging each year.^2^ Among various therapeutic interventions, anti‐seizure medications (ASMs) remain the primary treatment modality in clinical practice. Studies have found that the majority of patients could be successfully managed after using the first or second monotherapy medication.^3^, ^4^, ^5^ Additionally, adverse events of ASM monotherapy tend to be less frequent (and often less severe) due to the absence of adverse pharmacokinetic or pharmacodynamic interactions.^6^ Therefore, it is necessary to research the efficacy and safety of new‐generation anti‐seizure medications in monotherapy to provide clinicians with more medication options.

Perampanel (PER), approved by the US Food and Drug Administration (FDA) in 2012, stands out as the first selective noncompetitive α‐amino‐3‐hydroxy‐5‐methyl‐4‐isoxazolepropionic acid (AMPA) receptor antagonist. It binds to the postsynaptic membrane AMPA receptor, non‐competitively inhibiting the action of the excitatory neurotransmitter glutamate.^7^ In July 2021, PER received approval in China for both monotherapy and adjunctive treatment of focal onset seizures (FOS) with or without focal to bilateral tonic–clonic seizures (FBTCS) in adults and children over 4 years old.^8^ Subsequently, in 2023, PER was included as a first‐line medication for FOS in the clinical diagnosis and treatment guidelines of China (Epilepsy Volume).

Previous studies have yielded extensive clinical data on PER's efficacy and manageable adverse reactions as an adjunctive treatment in both adult and pediatric epilepsy.^9^, ^10^, ^11^, ^12^, ^13^ Given its novel mechanism of action and prolonged half‐life, there is growing interest among researchers in evaluating its efficacy and safety as monotherapy to expand clinical treatment options. Since its approval for use in China in 2021, numerous studies on PER have been undertaken. A recent study found that patients receiving PER monotherapy achieved superior seizure control compared to those on add‐on therapy.^14^ However, clinical data on PER monotherapy in pediatric epilepsy in China remain limited to small sample sizes (*N* < 100). Hence, we undertook this research to furnish clinical data on PER monotherapy in pediatric epilepsy treatment in China. Additionally, we tried to identify factors associated with therapeutic efficacy and explore populations better suited for PER monotherapy.

## 2 METHODS

### 2.1 Study design

This study was a real‐world clinical single‐center prospective observational study. We gathered real‐world clinical data of children with epilepsy who received PER as monotherapy at the Children's Hospital of Nanjing Medical University from October 2021 to October 2023. The study adhered to the principles outlined in the World Medical Association Declaration of Helsinki, and it received approval from the Ethics Committee of the Affiliated Children's Hospital of Nanjing Medical University (Approval number: 202309013‐1). All parents or guardians provided written informed consent for their children's participation in the study.

Follow‐up was conducted through various means, including the Neusoft medical software system, telephone calls, and online surveys. Upon enrollment, we collected baseline data, including demographics, age at seizure onset, seizure type, seizure frequency before medication, date and age at PER initiation, initial dose of PER (mg/day), maintenance dose of PER (mg/day), cranial magnetic resonance imaging, electroencephalography monitoring, and family history of epilepsy.

According to the drug instructions and literature reports, the administration regimen of PER for enrolled children was as follows: the initial dose of PER ranged from 0.5 to 2 mg/day, administered orally before bedtime each night. Dose titration was subsequently performed based on the child's response to epilepsy control, with an increase of 2 mg every 1–2 weeks. The maximum tolerated dose was determined based on the child's individual circumstances. Dose adjustments of PER were made according to patients' clinical responses and tolerability.

Inclusion criteria

1. Patients aged between 4 and 18 years old.
2. Patients diagnosed with childhood epilepsy according to the 2017 International League Against Epilepsy (ILAE) criteria.
3. Patients who had not received any prior anti‐seizure treatment.
4. Patients whose seizures were considered likely to be controlled with PER monotherapy by the treating physician based on the patient profile and clinical characteristics of epilepsy.

Exclusion criteria

1. Patients receiving add‐on therapy.
2. Patients undergoing secondary monotherapy, including conversion from adjunctive therapy to monotherapy by withdrawing concomitant ASMs or conversion from other ASMs monotherapy.
3. Patients with severe liver or kidney diseases.
4. Patients with lactose intolerance, lactose deficiency, or glucose–galactose malabsorption.

### 2.2 Data collection

During the follow‐up process, we closely monitored changes in seizure frequency, safety, and retention rate at 3, 6, 9, and 12 months after initiating PER monotherapy. Changes in seizure frequency and safety (adverse reactions) were documented based on physician evaluations, considering children's subjective experiences and their parents' daily observations. The decision about PER discontinuation was made according to best clinical practice.

### 2.3 Outcome measures

#### 2.3.1 Retention rate assessments

Retention rates were defined as the proportions of patients remaining on PER monotherapy at 3, 6, 9, and 12 months. All enrolled patients, excluding those lost to follow‐up and those who have not reached the required duration at enrollment, need to be analyzed.

#### 2.3.2 Efficacy assessments

Patients meeting the inclusion and exclusion criteria and receiving complete treatment for at least 3 months during the corresponding follow‐up period were included in the efficacy analysis set. This set encompassed seizure‐free, ≥50% but <100% seizure reduction, <50% seizure reduction and no change or even a worsening in seizure frequency. Seizure‐free referred to complete seizure control on PER monotherapy since the prior visit, while the ≥50% but <100% seizure reduction rate denoted a ≥50% reduction in seizure frequency every 3 months compared to baseline frequency and <50% seizure reduction meant a reduction in seizure frequency of less than 50% compared to baseline. Patients showing no change or worsening in seizure frequency did not respond to PER monotherapy. Patients discontinued from treatment before the 3‐month follow‐up point due to lack of response to PER, adverse effects, or loss to follow‐up were excluded from efficacy analysis. During subsequent follow‐ups, patients who discontinued monotherapy due to poor efficacy are still included in the analysis (equivalent to reaching the efficacy endpoint of PER monotherapy). Patients who discontinued monotherapy for other reasons (including loss to follow‐up and discontinuation due to side effects) are not included in the efficacy analysis set (as subsequent monotherapy efficacy data are missing). Seizure freedom rate was selected as the primary endpoint for our study.

#### 2.3.3 Safety assessments

Patients meeting the inclusion and exclusion criteria and receiving at least one PER treatment were included in the safety analysis set. Safety endpoints included the total number of patients experiencing at least one adverse reaction judged by the treating physician to be related to PER, the incidence of each adverse reaction, and adverse reactions resulting in discontinuation or dose reduction. Additionally, we evaluated whether adverse reactions improved after treatment adjustment.

### 2.4 Statistical analysis

Data analysis was conducted using IBM SPSS Statistics 23 software, with GraphPad Prism utilized for graphical representation. Quantitative variables were expressed as mean ± standard deviation or median (percentile), depending on data distribution, which was assessed using the Shapiro–Wilk test. Categorical data were presented as *n* (%). For single‐factor analysis, single‐factor logistic regression and Pearson's chi‐squared test were employed for qualitative data, with the continuity correction chi‐square test and Fisher's exact test used where appropriate. Multifactor analysis involved binary logistic regression to examine the influencing factors of efficacy. Statistical significance was set at *p* < 0.05.

## 3 RESULTS

### 3.1 Study population and baseline characteristics

A total of 124 patients (60 males and 64 females) were enrolled in the study. The average age at PER initiation was 8.25 ± 2.50 years, range from 4 to 15 years. Among them, 39 (31.45%) patients were younger than 7 years, while 85 (68.55%) patients were older than 7 years. The seizure onset age of these patients was 7.28 ± 2.52 years, ranging from 1.00 to 13.58 years. No statistically significant differences were observed in the age of seizure onset between males and females (*t* = 0.043, *p* = 0.966).

All patients received PER as monotherapy, serving as the primary anti‐seizure medication. The majority of patients presented with FOS (70.16%), while 2.42% had primary generalized tonic–clonic seizures (GTCS), and 27.42% had FBTCS. Table 1 outlines the baseline demographic and disease characteristics of the enrolled patients.

**TABLE 1:** Demographics and clinical features of the children.

Category	All patients (*N* = 124)
Age at PER initiation, mean ± SD (range)	8.25 ± 2.50 (4.00–15.00)
<7 years, *n* (%)	39 (31.45%)
≥7 years, *n* (%)	85 (68.55%)
Gender, *n* (%)	
Male	60 (48.39%)
Female	64 (51.61%)
Seizure onset age, mean ± SD (range)	7.28 ± 2.52 (1.00–13.58)
Baseline frequency 3 months before PER initiation Numbers, median (range)	2 (0–10)
Types of seizure *n* (%)	
FOS	87 (70.16%)
GTCS	3 (2.42%)
FBTCS	34 (27.42%)
Initial dose of perampanel (mg/day)	0.5–2.0
<7 years, median (IQR)	1.0 (1.0, 1.0)
≥7 years, median (IQR)	1.0 (1.0, 2.0)
Maintenance dose of perampanel (mg/day)	2.0–12.0
<7 years, median (IQR)	4.0 (3.0, 4.0)
≥7 years, median (IQR)	6.0 (4.0, 6.0)
MRI (*N* = 109), abnormal, *n* (%)	32 (29.36%)
EEG findings (*N* = 121), abnormal, *n* (%)	115 (95.04%)
Family history of epilepsy (*N* = 124), *n* (%)	19 (15.32%)

Abbreviations: FBTCS, focal to bilateral tonic–clonic seizures; FOS, focal onset seizures; GTCS, generalized tonic–clonic seizures; IQR, interquartile range; PER, perampanel; SD, standard deviation.

### 3.2 Retention rates

**FIGURE 1:**
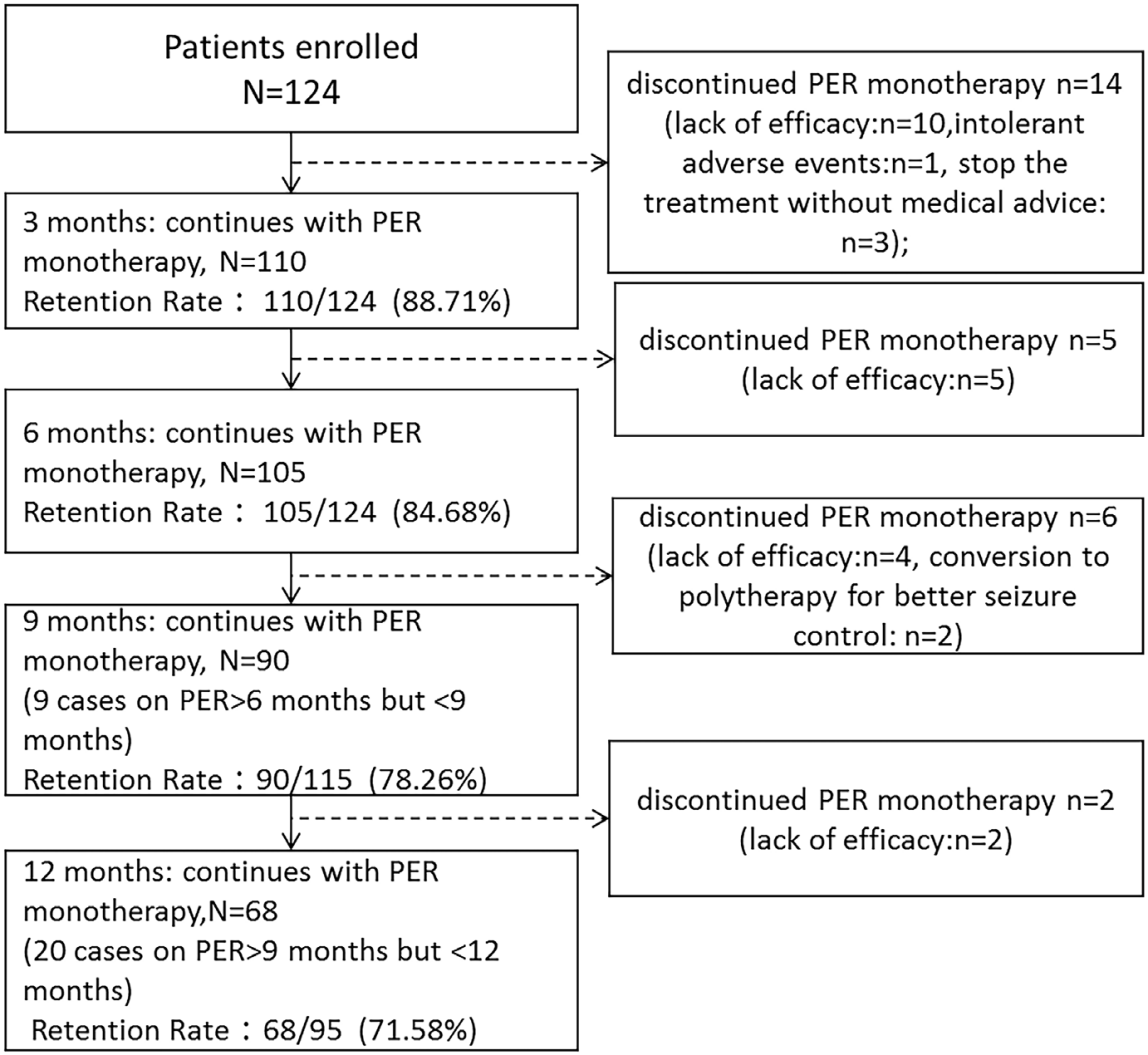
Flow Chart of patients at endpoint 3, 6, 9, and 12 months of follow‐up. PER, perampanel.

Figure 1 clearly shows the retention rates during the follow‐up period. By the end of the follow‐up, all 124 patients achieved a minimum follow‐up of 6 months. However, due to the study design (patients were enrolled at different times), 9 patients had not reached 9 months, and 20 had not reached 12 months. These patients were excluded from the respective retention rate analyses.

The overall retention rate at 3 months stood at 88.71% (110/124). Fourteen patients discontinued before this point. Ten discontinued due to lack of response, switching to adjunctive therapy, or secondary monotherapy with other ASMs. One discontinued due to intolerable adverse events, and three stopped without medical advice. At the 6‐month observation point (OP6), the retention rate decreased slightly to 84.68% (105/124), with five patients discontinuing due to lack of efficacy before OP6. The retention rate at 9 months was 78.26% (90/115) (nine patients continued PER monotherapy treatment for more than 6 months but less than 9 months). Before OP9, six patients discontinued PER monotherapy, with four doing so due to lack of efficacy, and the other two choosing to switch to polytherapy for better seizure control, despite achieving ≥50% but <100% seizure reduction during the PER monotherapy follow‐up period. By 12 months, the retention rate was 71.58% (68/95) (twenty patients continued PER monotherapy treatment for more than 9 months but less than 12 months). Two patients discontinued PER monotherapy treatment due to lack of efficacy before OP12.

### 3.3 Clinical efficacy

**FIGURE 2:**
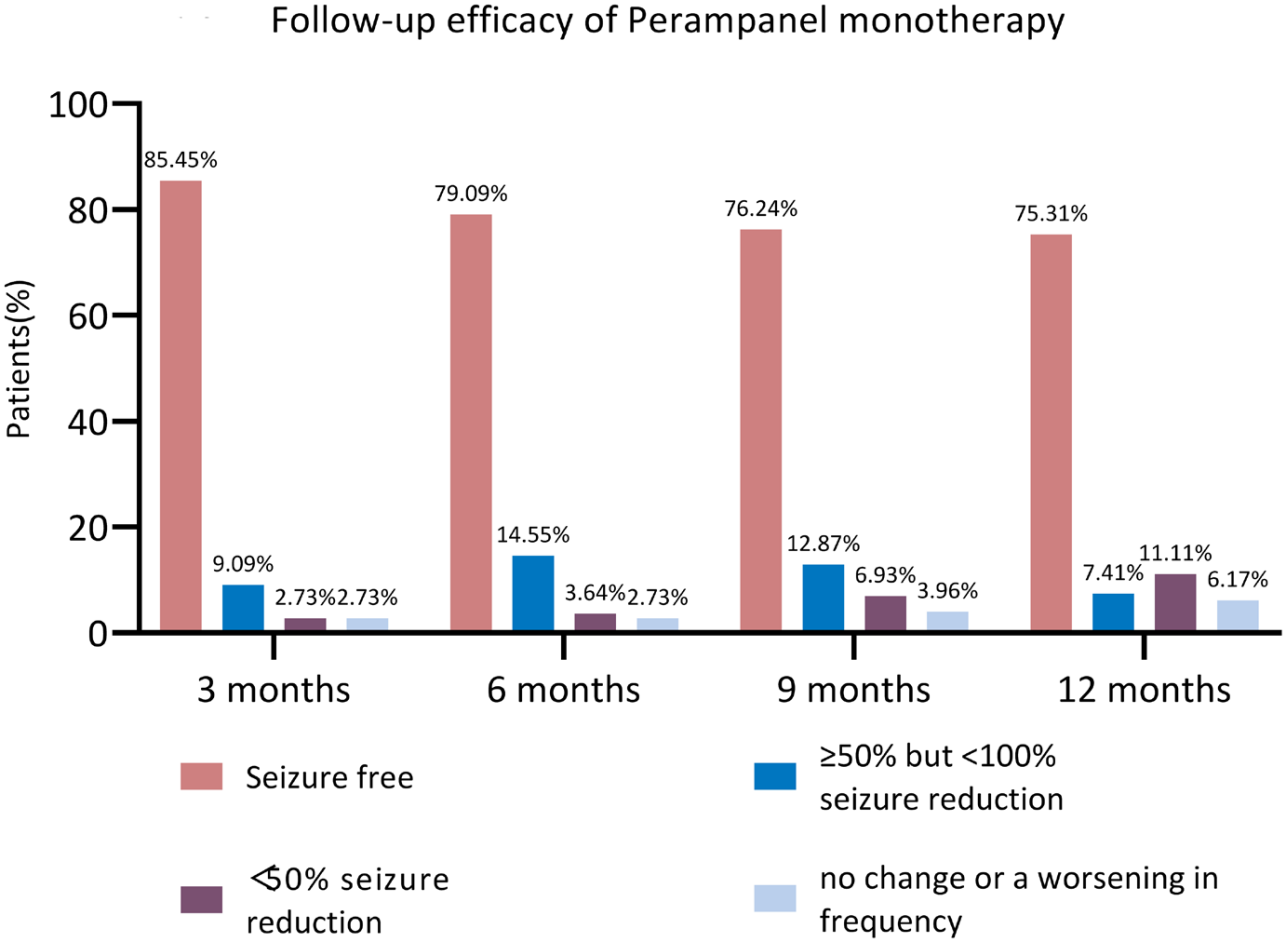
Follow‐up efficacy of Perampanel monotherapy.

The efficacy analysis set comprised a total of 110 patients. The seizure‐free rates at 3, 6 months, 9, and 12 months were 85.45% (94/110), 79.09% (87/110), 76.24% (77/101), and 75.31% (61/81), respectively. Additionally, the rates of >50% but <100% seizure reduction were 9.09% (10/110), 14.55% (16/110), 12.87% (13/101), and 7.41% (6/81) for the same respective time points (Figure 2).

Factors involved in the efficacy analysis included age at PER initiation, seizure onset age, gender, baseline frequency, seizure types, duration of treatment, family history of epilepsy, and maintenance dose. Among these factors, duration of treatment (OR = 8.090, 95% CI 2.319–28.217, *p* = 0.001) and maintenance dose (OR = 0.716, 95% CI 0.537–0.954, *p* = 0.022) significantly influenced the efficacy of PER monotherapy (Table 2, Table S2).

**TABLE 2:** Univariate analysis of effective rates of different types of perampanel monotherapy.

Variables	Total (110)	<100% seizure reduction rate	Seizure freedom rate	*Χ* ^2^/OR (95% CI)	*p* value
Age at perampanel initiation^b^	110	19 (17.3%)	91 (82.7%)	0.972 (0.795–1.188)	0.780
Seizure onset age^b^	110	19 (17.3%)	91 (82.7%)	0.970 (0.784–1.199)	0.775
Gender^a^					
Male	50	12 (24.0%)	38 (76.0%)	2.903	0.088
Female	60	7 (11.7%)	53 (88.3%)		
Baseline frequency^b^	110	19 (17.3%)	91 (82.7%)	0.766 (0.617–0.952)	0.016
Seizure types^a^					
FBTCS/GTCS	30	6 (20.0%)	24 (80.0%)	0.215	0.643
FOS	80	13 (16.3%)	67 (83.8%)		
Duration of treatment^c^					
3 m/6 m	20	9 (45.0%)	11 (55.0%)	10.887	0.001
9 m/12 m	90	10 (11.1%)	80 (88.9%)		
Family history of epilepsy^c^					
No	95	15 (15.8%)	80 (84.2%)	0.446	0.504
Yes	15	4 (26.7%)	11 (73.3%)		
Maintenance dose^b^	110	19 (17.3%)	91 (82.7%)	0.745 (0.585–0.947)	0.016

^a^ Pearson's chi‐squared test.

^b^ Binary logistic regression.

^c^ Continuity correction chi‐square test.

### 3.4 Adverse effects and safety

Safety data were exclusively discussed for patients included in the safety analysis sets, defined as those with available safety data who received PER monotherapy. In our study, 124 patients had safety data available and were therefore included in the safety analysis sets. Among them, 48 patients (38.7%) experienced at least one adverse effect. Some of these patients experienced two or more adverse effects, with the most common being irritability in 19 cases (15.3%), followed by dizziness in 18 cases (14.5%). The incidence of other adverse effects was all below 5%. Notably, one patient withdrew within 1 month due to unbearable itching of the skin. No other serious adverse effects significantly associated with PER treatment were observed in our study.

Factors involved in the safety analysis set also included age at PER initiation, seizure onset age, gender, baseline frequency, seizure types, duration of treatment, family history of epilepsy, and maintenance dose. Among these factors, the incidence of adverse effects was statistically significant between males and females (refer to Table 3, Table S3). The incidences of every treatment‐emergent adverse event (TEAEs) in our study are shown in Table 4.

**TABLE 3:** Univariate analysis of adverse effects of PER monotherapy.

Variables	Total (124)	Patients with any adverse effects	*Χ* ^2^/OR (95% CI)	*р* value
Age at perampanel initiation^b^	124	48 (38.7%)	1.171 (1.008–1.360)	0.039
Seizure onset age^b^	124	48 (38.7%)	1.134 (0.979–1.314)	0.094
Gender^a^				
Male	60	30 (50.0%)	6.246	0.012
Female	64	18 (28.1%)		
Baseline frequency^b^	124	48 (38.7%)	1.170 (1.004–1.365)	0.045
Seizure types^a^				
FBTCS/GTCS	37	13 (35.1%)	0.284	0.594
FOS	87	35 (40.2%)		
Duration of treatment^c^				
<3 m	14	6 (42.9%)	4.700	0.322
3 m	5	4 (80.0%)		
6 m	15	6 (40.0%)		
9 m	22	6 (27.3%)		
12 m	68	26 (38.2%)		
Family history of epilepsy^a^				
No	105	42 (40.0%)	0.481	0.488
Yes	19	6 (31.6%)		
Maintenance dose^b^	124	48 (38.7%)	1.227 (0.998–1.509)	0.052

^a^ Pearson's chi‐squared test.

^b^ Binary logistic regression.

^c^ Fisher's exact test.

**TABLE 4:** Incidence of adverse effects.

	Total	Gender cohort		*p* ^a^
	*N* (%)	Male, *n* (%)	Female, *n* (%)	
	*N* = 124	*N* = 60	*N* = 64	
	*n* = 48 (38.7)	*n* = 30 (50.0)	*n* = 18 (28.1)	
Dizziness	18 (14.5)	11 (18.3)	7 (10.9)	0.243
Somnolence	5 (4.0)	2 (3.3)	3 (4.7)	1.000
Ataxia	3 (2.4)	2 (3.3)	1 (1.6)	0.955
Depression	1 (0.8)	1 (1.7)	0 (0.0)	–
Headache	2 (1.6)	1 (1.7)	1 (1.6)	1.000
Weight gain	4 (3.2)	3 (5.0)	1 (1.6)	0.566
Nausea	2 (1.6)	2 (3.3)	0 (0.0)	–
Irritability	19 (15.3)	13 (21.7)	6 (9.4)	0.058
Skin itching	1 (0.8)	1 (1.7)	0 (0.0)	–
Increased appetite	4 (3.2)	3 (5.0)	1 (1.6)	0.566
Enuresis	4 (3.2)	2 (3.3)	2 (3.1)	1.000

^a^ Pearson's chi‐squared test OR Continuity correction chi‐squared test OR Fisher's exact test.

## 4 DISCUSSION

An analysis of real‐world clinical data on PER primary monotherapy(PM) was conducted in a relatively large cohort of 124 Chinese pediatric patients. The study demonstrated an impressive seizure freedom rate of over 70%, along with a retention rate of 71.58% at the 12‐month observation point (OP12).

Phase III clinical trial (Study 342 [FREEDOM]), the first study to investigate PER monotherapy to patients with newly diagnosed epilepsy, reported seizure freedom rates ranging from 63.0% to 74.0% at the 26‐week Maintenance Period.^15^ A multicenter real‐world study in Europe,^16^ with a smaller number of patients with PM, reported seizure freedom rates of PER PM were 71.4% at 6 months and 58.3% at 12 months. These studies above demonstrated initial feasibility of PER PM. Takuji Nishida et al. further reported similar response rate in Asian and non‐Asian populations.^17^

Comparing our findings to existing research about Asian populations, the seizure freedom rate observed in our study aligns well with results from other prospective studies and clinical trials evaluating PER monotherapy (Seizure freedom rate at OP12: 52%–83%).^18^, ^19^, ^20^, ^21^, ^22^ A prospective study of PER monotherapy in patients (aged 34.11 ± 17.19 years old) with FOS demonstrated seizure freedom rates of 69.84% and 65.08% at different observation points.^23^ The seizure freedom rate of patients aged 4–18 years in this research^23^ was 84.21% (16/19), 73.68% (14/19) at OP6, OP12, which was consistent with the seizure freedom rate in our study (79.09%, 75.31% at OP6, OP12). Furthermore, a retrospective analysis of PER monotherapy in pediatric patients with self‐limited epilepsy with centrotemporal spikes (SeLECTs)^20^ revealed high seizure freedom rates exceeding 97% initially and gradually declining over time. Our study extends these efficacy findings by including a substantial number of patients aged 4–18 years, reinforcing the effectiveness of PER monotherapy across this pediatric age range.

Furthermore, our study revealed that the duration of treatment with PER monotherapy significantly impacted its efficacy. Patients who continued monotherapy for 9 months or longer achieved better seizure control and higher seizure‐free rates compared to those who adhered to treatment for 6 months or less. Besides, six of these patients had <50% seizure reduction at the 3‐month OP, but with dose escalation, three of them achieved over 50% seizure reduction at 9 months OP and beyond. Decisions to discontinue or switch from perampanel monotherapy may not be solely based on seizure response before reaching an effective dose.^24^ A study on early identification of refractory epilepsy^4^ found that nearly half of the patients achieved seizure freedom with the first ASM, emphasizing the significance of the initial treatment choice. Moreover, patients who discontinued the first‐line treatment due to lack of efficacy had notably lower rates of seizure freedom with subsequent therapies, indicating the potential impact of early treatment decisions on long‐term outcomes. The findings above emphasize the importance of early initiation and sustained adherence to PER monotherapy under the guidance of a physician to achieve optimal seizure control in pediatric patients with epilepsy.

The observation that patients on PER monotherapy with a relatively low maintenance dose have a higher seizure‐free rate is an intriguing finding that warrants careful consideration. In our study, conducted in a real clinical setting, drug doses were adjusted promptly based on efficacy, with close attention to balancing efficacy and safety. In cases where seizure control was achieved after dose escalation, an attempt to reduce the dose by 2 mg/day may have been implemented to reduce adverse events. If the patient maintained good seizure control after the dose reduction, the dose was kept unchanged. This approach aligns with recommendations from a novel review on expert opinion in Asia^25^ regarding PER dose optimization, which suggests adjusting the maintenance dose according to age and titrating the dose based on the patient's response. For our study population consisting of children aged 4–18 years newly diagnosed with epilepsy, maintaining the dose after achieving seizure freedom can be considered during the process of adjusting PER monotherapy. However, it is crucial to emphasize that dose adjustment should follow the principles of clinical individualization, with a focus on optimizing efficacy while minimizing adverse events. Blindly pursuing high doses without regard for individual patient response may not be beneficial and could potentially lead to unnecessary side effects. Therefore, a tailored approach to dose adjustment based on ongoing clinical assessment and patient response is essential for optimizing the therapeutic benefit of PER monotherapy in pediatric epilepsy patients.

A systematic review and network meta‐analysis, which encompassed a substantial number of randomized controlled trials (RCTs) and patients, highlighted PER as the best‐ranked therapy in terms of tolerability compared to other ASMs, whether used as monotherapy or adjunctive therapy.^26^ In our study, most of adverse effects observed were mild to moderate, which were comparable with that had reported.^27^ Withdrawal from treatment due to adverse events was rare, with only one case attributed to unbearable itching of the skin, suggesting a generally favorable tolerability profile. Enuresis, observed in four cases in our study, warrants further investigation to determine its potential correlation with PER use, as sporadic reports of this adverse event have been noted in earlier studies.^21^, ^28^ Additionally, while psychiatric or behavioral disorders such as irritability and depression were observed in a subset of patients in our series, none discontinued PER monotherapy due to these issues. Long‐term follow‐up observations indicated that irritability tended to diminish over time without leading to severe consequences, similar to previous studies.^19^, ^29^, ^30^ It is essential to recognize the impact of perceptions of stigma on pediatric patients' emotions and not solely attribute emotional issues to medication, especially in older children.^31^

In our study, the probability of any one adverse reaction occurring is higher in males than females, with males being 2.268 times more likely than females. Specifically and for instance, the proportion of male patients experiencing dizziness was numerically higher than females, but it did not reach statistical significance (*p* = 0.243). A subanalysis of phase III randomized clinical studies enrolled male and nonpregnant female subjects ≥12 years of age and found that the incidence of headache and dizziness is higher in females (31.5%) than in males (24.4%),^32^ which both numerically higher than the incidence in our study (females 10.9%; males 18.3%). Although our study seemed to show opposite gender ratios in the occurrence of dizziness and headache compared to this previous study, the difference did not reach statistical significance. Further research in larger populations is warranted to explore potential gender‐based differences in adverse event profiles associated with perampanel monotherapy. Overall, our findings support PER's favorable tolerability profile, making it a valuable treatment option for patients with epilepsy, particularly in cases where other ASMs may pose tolerability challenges. However, ongoing monitoring and research are essential to better understand and manage potential adverse effects associated with PER use.

Our study also has several limitations. This was a real‐world single‐line prospective observational study and did not include a control group. Besides, in line with other real‐world observational studies, TEAEs were only reported if they had been recorded in patient notes during routine clinical care, which may have led to unstandardized evaluation of TEAEs in our study. Future studies on the efficacy and safety of PER require more RCTs and larger sample sizes. We expect more multicenter real‐world studies of PER monotherapy in children to contribute efficacy and safety data.

## 5 CONCLUSIONS

We demonstrated the efficacy and safety of PER monotherapy as primary anti‐seizure medication in the treatment of children's epilepsy. This study is the largest single‐center monotherapy study of PER on children in terms of sample size in China to date. Patients who respond well to this drug and adhere to long‐term treatment can achieve favorable seizure control. Besides, patients who achieve seizure freedom with a relatively lower dose can choose the same dose as the maintenance dose. High dosage is not the treatment goal; Achieving effective and safe seizure control is the treatment objective, and dosage selection should be personalized.

## AUTHOR CONTRIBUTIONS

*Concept and design*: Jing Chen, Feng Chen, and Wu. *Data collection and analysis*: Gu, Wei Li, and Yue Li. *Drafting of the manuscript*: Gu, Wei Li, and Jing Chen. *Critical revision of the manuscript*: Jing Chen. *Study supervision*: Jing Chen and Wu. *Recruitment of patients into study and follow‐up*: Jing Chen and Gu. All authors approved the submitted version of the manuscript.

## FUNDING INFORMATION

This study did not receive any specific grant from funding agencies in the public, commercial, or not‐for‐profit sectors.

## CONFLICT OF INTEREST STATEMENT

None of the authors has any conflict of interest to disclose. We confirm that we have read the Journal's position on issues involved in ethical publication and affirm that this report is consistent with those guidelines.

## PATIENT CONSENT STATEMENT

The study adhered to the principles outlined in the World Medical Association Declaration of Helsinki, and it received approval from the Ethics Committee of the Affiliated Children's Hospital of Nanjing Medical University (Approval number: 202309013–1). All parents or guardians provided written informed consent for their children's participation in the study.

## Supporting information


Data S1.

## Data Availability

Data can be accessible to other researchers through a reasonable request.
