# Supplementary material for: Clinical efficacy and safety of perampanel monotherapy as primary anti‐seizure medication in the treatment of pediatric epilepsy: A single‐center, prospective, observational study
Source: Epilepsia Open. 2024 Sep 18;9(6):2209–18. doi: 10.1002/epi4.13043 (PMC11633688; doi:10.1002/epi4.13043)
Supplement: Supplementary file 1 — Data S1. [file EPI4-9-2209-s001.docx]

**Supplementary file**

TABLE S1: Efficacy of perampanel monotherapy at different observation points

| Observation point | total | Seizure free | ≥50% but <100% seizure reduction | ＜50% seizure reduction | no change or a worsening in frequency |
| --- | --- | --- | --- | --- | --- |
| 3 months | 110 | 94 (85.45%) | 10 (9.09%) | 3 (2.73%) | 3 (2.73%) |
| 6 months | 110 | 87 (79.09%) | 16 (14.55%) | 4 (3.64%) | 3 (2.73%) |
| 9 months | 101 | 77 (76.24%) | 13 (12.87%) | 7 (6.93%) | 4 (3.96%) |
| 12 months | 81 | 61 (75.31%) | 6 (7.41%) | 9 (11.11%) | 5 (6.17%) |

TABLE S2: Multivariate analysis of contributing factors of efficacy of perampanel monotherapy

| **Variables** | *b* value | $S_{b}$ | Wald χ^2^ | *р* value | Odds ratio | 95%CI Of OR |
| --- | --- | --- | --- | --- | --- | --- |
| **Gender** | 0.809 | 0.604 | 1.792 | 0.181 | 2.245 | 0.687-7.337 |
| **Baseline frequency** | -0.183 | 0.124 | 2.160 | 0.142 | 0.833 | 0.653-1.063 |
| **Seizure types** | 0.173 | 0.666 | 0.068 | 0.795 | 1.189 | 0.322-4.389 |
| **Duration of treatment** | 2.091 | 0.637 | 10.758 | 0.001 | 8.090 | 2.319-28.217 |
| **Maintenance dose** | -0.334 | 0.146 | 5.214 | 0.022 | 0.716 | 0.537-0.954 |

TABLE S3: Multivariate analysis of contributing factors of adverse effects of PER monotherapy

| Variables | group | b value | $S_{b}$ | Wald χ2 | р value | Odds ratio | 95%CI Of OR |
| --- | --- | --- | --- | --- | --- | --- | --- |
| age at PER initiation |  | 0.102 | 0.122 | 0.698 | 0.404 | 1.108 | 0.871-1.408 |
| Seizure onset age |  | 0.034 | 0.116 | 0.084 | 0.771 | 1.034 | 0.823-1.299 |
| Gender | Male | 0.819 | 0.396 | 4.275 | 0.039 | 2.268 | 1.044-4.929 |
|  | Female* |  |  |  |  |  |  |
| Baseline frequency |  | 0.123 | 0.084 | 2.136 | 0.144 | 1.131 | 0.959-1.334 |
| Maintenance dose |  | 0.088 | 0.120 | 0.545 | 0.460 | 1.092 | 0.864-1.382 |

*: control groups;

We approached the assessment of PER primary monotherapy efficacy from two distinct angles: follow-up efficacy and factors influencing efficacy. These perspectives offer nuanced insights into treatment outcomes. Follow-up efficacy primarily involves tracking changes in efficacy at different observation points throughout the long-term follow-up period for all patients included in the efficacy analysis set. By examining variations in efficacy over time, we gain a comprehensive understanding of how PER monotherapy performs throughout the treatment duration. In contrast, the analysis of factors influencing efficacy involves considering various independent variables, with treatment duration being one of them. We assessed the final follow-up efficacy for each patient's follow-up period to evaluate how different factors impact treatment outcomes. This approach allows us to identify factors that may influence the efficacy of PER monotherapy, providing valuable insights into treatment success determinants. These two analytical perspectives complement each other, offering a comprehensive view of PER primary monotherapy efficacy and contributing to a deeper understanding of its effectiveness in treating pediatric epilepsy.
